# Seasonality and clinical characteristics of influenza-like illness and severe acute respiratory infection in children under 5 years in Cox’s Bazar, Bangladesh (2021-2023)

**DOI:** 10.1016/j.ijregi.2026.100921

**Published:** 2026-05-22

**Authors:** Md. Osman Hossain Zabir, Md. Ahashan Habib, Monalisa, Md. Mahbubur Rahman, Alif Nur Ayman, Quazi Zaki Ahmed, Urmila Rahman, Rakib Hossain, Tahmina Shirin

**Affiliations:** 1 Directorate General of Health Service, Dhaka, Bangladesh; 2 Mycobacterial Disease Control, Directorate General of Health Services, Ministry of Health and Family Welfare, Bangladesh; 3 Institute of Epidemiology, Disease Control & Research, Mohakhali, Dhaka, Bangladesh; 4 Dhaka Medical College Hospital. Dhaka, Bangladesh; 5 Eastern Medical College, Cumilla, Bangladesh; 6 Dr. Sirajul Islam Medical College & Hospital Ltd., Dhaka, Bangladesh; 7 Khulna University, Khulna, Bangladesh

**Keywords:** Influenza, Seasonality, Clinical severity indicators, Surveillance, Bangladesh, Cox’s Bazar

## Abstract

**Objectives:**

Acute respiratory infections (ARIs) such as influenza-like illness (ILI) and severe acute respiratory infection (SARI) are leading causes of morbidity among children aged under 5 years (under-5) in Bangladesh. In Cox’s Bazar, the ARI burden is compounded by high population density and seasonal monsoons; yet under-5–specific evidence on virus seasonality and clinical features remains limited. This study aimed to assess the seasonality and clinical characteristics of ILI and SARI among children under-5 in Cox’s Bazar, Bangladesh, between 2021 and 2023, with additional analyses to support interpretation of detection findings.

**Methods:**

Prospective hospitalized based surveillance was carried out from January 2021 up to December 2023 at the District Head Quarter Hospital Cox’s Bazar. Children under-5 with ILI (fever ≥38°C and a cough, symptom onset ≤10 days) or SARI (ILI with hospitalization or severe manifestations) were recruited. Nasopharyngeal and throat swabs were processed for testing by a multiplex real-time reverse-transcriptase polymerase chain reaction for influenza A/B (H3N2/Victoria), SARS-CoV-2, and respiratory syncytial virus (RSV). Demographic, clinical, and geographic information was recorded. Analyses included descriptive statistics, chi-square tests, and multivariable logistic regression.

**Results:**

Among 968 children (median age 9 months; 60.7% ILI, 39.3% SARI), pathogens were detected in 12.4% (95% confidence interval [CI]: 10.4-14.7%). Among the limited pathogens tested, influenza comprised most positive cases (65%); A(H3N2) was identified in 5.0%, and B(Victoria) in 3.1%. SARS-CoV-2 and RSV were detected only occasionally (2.5% and 1.9%, respectively). The monsoon season (June-September) was the peak detection period, with A(H3N2) being dominant in June and July and B(Victoria) dominating from August to September. Positivity was higher among SARI cases (25.8%) than ILI cases (16.4%) in the months of peak activity. Clinical severity indicators associated with detection were SARI (adjusted odds ratio [aOR] 3.42, 95% CI: 2.28-5.13), breathlessness (aOR 2.87, 95% CI: 1.84-4.48), age ≥6 months to <2 years (aOR 1.92, 95% CI:1.21-3.05) and fever ≥101.5°F (aOR 1.68, 95% CI: 100-276). The highest positivity rate observed was 24.0%. Most (87.6%) were negative for the tested pathogens.

**Conclusion:**

Among the viruses tested, influenza contributes to seasonal under-5 ARIs in Cox’s Bazar, but the large negative proportion indicates that most cases were caused by pathogens not included in the limited testing panel (e.g. rhinovirus, adenovirus, parainfluenza, or bacteria). Markers of severity and age are associated with influenza positivity. Expanded multiplex testing and surveillance are required to inform vaccination and interventions in this high burden setting.

## Introduction

Acute respiratory infections (ARIs), including influenza-like illness (ILI) and severe ARI (SARI), continue to be a leading cause of morbidity and mortality among children aged under 5 years (under-5) worldwide, particularly, in low- and middle-income countries [1]. Worldwide, ARIs (primarily pneumonia) account for a substantial proportion of under-5 deaths, with infectious diseases such as pneumonia playing a major role alongside preterm birth complications and other preventable causes [1]. In 2023, an estimated 4.8 million children under-5 died worldwide, equivalent to approximately 13,100 deaths per day, with the global under-5 mortality rate at 37 deaths per 1000 live births—a 61% decline since 1990 but still far from the Sustainable Development Goal target of ≤25 by 2030 [2]. Recent projections indicate a worrying reversal: for the first time this century, under-5 deaths may rise in 2025 (to ∼4.8 million), driven partly by funding cuts to humanitarian and development aid, which may aggravate preventable mortality in vulnerable settings [3].

ARIs remain a significant health challenge for young children in Bangladesh, with pneumonia accounting for approximately 13-15% of under-5 mortality according to recent estimates [4]. National data indicate ongoing challenges, including a high prevalence of ARI symptoms among children under-5, which ranges from 20% to 65% in community surveys, depending on region and season. These rates are influenced by factors such as household wealth, overcrowding, and limited access to health care services [5]. Respiratory viruses, including influenza and respiratory syncytial virus (RSV), are major contributors to this burden. Hospital-based surveillance conducted by the Institute of Epidemiology, Disease Control and Research (IEDCR) and its partners has consistently identified influenza positivity in 10-20% of tested ILI and SARI cases and RSV in up to 30-36% of severe under-5 hospitalizations between 2022 and 2024 [6,7]. Distinct seasonal patterns are observed, with influenza peaking during the monsoon season (June-September) and other viruses exhibiting variable circulation. Broader pathogen panels further increase detection rates, particularly, when rhinovirus, adenovirus, and parainfluenza viruses are included [8].

Cox’s Bazar District in south-eastern Bangladesh experiences distinct environmental and demographic challenges that increase the risk of ARIs, such as seasonal monsoons, flooding, and high population density in specific upazilas [9]. Health reports from the district consistently identify ARIs as leading contributors to under-5 morbidity, with notable increases during the monsoon season [10]. However, a specific knowledge gap remains: although national surveillance provides aggregate data, under-5–specific evidence on virus seasonality and clinical features in Cox’s Bazar has been sparse since 2020 due to COVID-19–related disruptions and resource limitations [11]. Furthermore, existing surveillance uses limited testing panels, creating a substantial etiological gap [12]. This study addresses these gaps by describing the seasonality and clinical features associated with viral detection in under-5 ILI and SARI cases enrolled at the 250 Bedded District Hospital in Cox’s Bazar from 2021 to 2023 while acknowledging the limitations of a narrow pathogen panel and aiding interpretation of findings.

## Methods

### Study design and setting

A prospective, hospital-based surveillance study was conducted from January 1, 2021 to December 31, 2023, at the 250 Bedded District Hospital in Cox’s Bazar, Bangladesh. This hospital served as the primary sentinel site for pediatric respiratory surveillance in the district, receiving patients from outpatient departments (OPDs) and inpatient wards (medicine and pediatrics). This study was conducted within the framework of the National Influenza Surveillance Bangladesh, a hospital-based influenza surveillance (HBIS) system coordinated by the IEDCR in collaboration with the World Health Organization (WHO). The surveillance operated in strict alignment with the WHO Global Influenza Surveillance and Response System (GISRS) standards. A dedicated surveillance team, comprising two physicians, two nurses, and two medical technologists recruited from existing hospital staff, was responsible for case identification, enrollment, specimen collection, and follow-up. A project facilitator coordinated site activities and acted as the primary contact for the IEDCR.

### Study population and case definitions

Children under-5 presenting to the pediatric OPDs or inpatient wards with symptoms meeting the definitions of ILI or SARI were enrolled. Case definitions adhered to WHO standards as adapted in Bangladesh’s national influenza surveillance protocols during the study period.

○ ILI was defined as an ARI with measured fever ≥38°C and cough, with onset within the last 10 days (outpatient presentation).
○ SARI was defined as an ARI with a history of fever or measured fever ≥38°C and cough, with onset within the last 10 days, and requiring hospitalization (or for children under-5, clinical signs of severe illness such as breathlessness, tachypnoea, or chest indrawing).

Exclusion criteria were symptom onset more than 10 days before presentation, non-residency in Cox’s Bazar District, or refusal of informed consent by the guardian. For ILI, systematic sampling targeted up to eight pediatric cases per week from the OPD, balanced between medicine and pediatric departments, where applicable. For SARI, all eligible admitted cases in the pediatric wards were enrolled. Surveillance physicians and nurses performed daily screening by reviewing admission records and examining patients to identify cases meeting the study definitions [6].

### Data collection

Trained surveillance team members used standardized case report forms adapted from national surveillance tools to collect data on sociodemographic characteristics, including age (categorized as <6 months, 6 months to <2 years, and 2-5 years), sex, and upazila of residence. Clinical information included presenting symptoms such as fever, cough, breathlessness, and vomiting, along with vital signs (temperature in°F) and relevant comorbidities, including asthma and chronic respiratory disease. Information on potential risk factors, such as exposure history (e.g. recent travel), case classification (ILI vs SARI), and date of symptom onset, was also recorded. For SARI cases, outcomes were monitored throughout hospitalization and documented monthly. In addition, aggregate monthly data, including total admissions, number of SARI cases, and deaths in surveillance wards, were collected to support denominator-based analyses. Data entry was performed using a secure electronic database with integrated validation features. Monthly summary reports were generated to provide denominators for the surveillance population.

### Specimen collection and laboratory testing

Trained medical technologists collected throat and nasal swabs from enrolled children within 10 days of symptom onset. Swabs were placed in separate containers with viral transport medium and subsequently pooled for testing. Specimens were initially stored at 2-8°C for up to 72 hours after collection, then transferred to dry shippers containing liquid nitrogen (approximately −196°C) for longer-term storage if necessary. Samples were transported weekly to the IEDCR National Influenza Centre laboratory in Dhaka by designated personnel, using dry shippers refilled with liquid nitrogen or cold boxes with ice packs within 48 hours when required.

Specimens underwent multiplex real-time reverse transcription polymerase chain reaction testing for the following:

○ Influenza A (with H3N2 subtyping),
○ Influenza B (Victoria lineage),
○ SARS-CoV-2, and
○ RSV.

No other respiratory viruses (e.g. rhinovirus, adenovirus, parainfluenza, human metapneumovirus) or bacterial pathogens were tested.

Laboratory testing was conducted according to standardized protocols established by the IEDCR, Bangladesh’s National Influenza Centre, in alignment with the WHO GISRS guidelines [6]. Testing was conducted using Centers for Disease Control and Prevention or equivalent primers and probes, with a cycle threshold value below 35 considered positive. Quality control measures included extraction controls, positive and negative controls, and internal amplification controls. Laboratory turnaround time was monitored monthly, and improvements were observed throughout the study period.

### Statistical analysis

Data analysis was performed using SPSS version 26 and R version 4.2 or later. Descriptive statistics included frequencies with percentages and 95% confidence intervals (CIs) where appropriate, medians with interquartile ranges (IQRs), and means with SDs.

A hypothesis-driven approach was used: we first compared detection rates by case type, age, and season using chi-square tests. Multivariable logistic regression was then performed to identify clinical features independently associated with viral detection, adjusting for *a priori* confounders (age, sex). Model fit was assessed via Nagelkerke R² and the Hosmer–Lemeshow test. Temporal trends were evaluated using chi-square tests for trend. Autoregressive integrated moving average modeling was explored but not retained due to insufficient data points for robust seasonal decomposition; therefore, only descriptive seasonal patterns are reported. Statistical significance was set at *P* <0.05 (two-tailed).

### Ethical considerations

The study was conducted in accordance with the Declaration of Helsinki and approved by the Institutional Review Board of the IEDCR (Protocol: Surveillance and Response to Avian and Pandemic Influenza, Memo: IEDCR/IRB/2025/13). Written informed consent was obtained from the parents or legal guardians of all children before enrollment. The surveillance team ensured that the rights and welfare of all participants were protected throughout the study.

## Results

### Study population demographics and clinical characteristics

Between January 2021 and December 2023, a total of 968 under-5 patients presenting with ILI and SARI were enrolled in a prospective surveillance study in Cox’s Bazar District, Bangladesh. The cohort comprised 588 ILI cases (60.7%) and 380 SARI cases (39.3%). The study population was characterized by young age, with a median age of 9 months (IQR: 4-24 months). As shown in Table 1, infants aged under 6 months constituted 32.2% of the study population (n = 312), followed by children aged 6 months to <2 years (n = 388, 40.1%) and those aged 2-5 years (n = 268, 27.7%). The sex distribution showed a male predominance (n = 580, 59.9% male; n = 388, 40.1% female), with the chi-square test revealing no significant association between sex and age group (χ² = 2.15, *P* = 0.341).

**Table 1:** Demographic and clinical characteristics of under-5 ILI and SARI cases (N = 968).

Characteristic	Category	Frequency	Percentage	95% CI	Test statistic
**Case type**	ILI	588	60.7%	57.6-63.8%	
	SARI	380	39.3%	36.2-42.4%	
**Age group**	<6 months	312	32.2%	29.3-35.3%	χ² = 3.24, *P* = 0.198^a^
	6 months to <2 years	388	40.1%	37.0-43.2%	
	2-5 years	268	27.7%	24.9-30.6%	
**Sex**	Male	580	59.9%	56.8-63.0%	
	Female	388	40.1%	37.0-43.2%	
**Geographic distribution**	Sadar	245	25.3%	22.6-28.2%	χ² = 34.21, *P* <0.001
	Ramu	128	13.2%	11.1-15.6%	
	Chakaria	112	11.6%	9.6-13.8%	
	Ukhia	98	10.1%	8.3-12.2%	
	Teknaf	87	9.0%	7.3-11.0%	
	Naikonchori	75	7.7%	6.1-9.7%	
	Moheshkhali	65	6.7%	5.2-8.5%	
	Pekua	58	6.0%	4.6-7.7%	
	Other	100	10.3%	8.5-12.4%	
**Clinical presentation**	Fever	928	95.9%	94.4-97.0%	
	Cough	905	93.5%	91.7-94.9%	
	Breathlessness	186	19.2%	16.8-21.8%	
	Vomiting	124	12.8%	10.8-15.1%	
**Vital signs (mean ± SD)**	Temperature (°F)	101.8 ± 1.2		101.7-101.9	t = 1.87, *P* = 0.062^b^
	Male: 101.8 ± 1.1				
	Female: 101.9 ± 1.3				
**Comorbidities**	Asthma	112	11.6%	9.6-13.8%	
	Chronic respiratory disease	48	5.0%	3.7-6.6%	
**Exposure history**	Travel	78	8.1%	6.4-10.0%	

CI, confidence interval; ILI, influenza-like illness; RSV, respiratory syncytial virus; SARI, severe acute respiratory infection.

a Chi-square test for association between sex and age group.

b Independent samples *t*-test for temperature difference by sex.

### Etiological spectrum and pathogen detection rates—limited panel

The overall pathogen detection rate was 12.4% (n = 120 of 968; 95% CI: 10.4-14.7%). Given the limited panel (influenza A/B, SARS-CoV-2, RSV only), this low rate is expected. A binomial test confirmed this proportion was significantly lower than the expected 50% (*P* <0.001). Table 2 presents the distribution of detected pathogens. Among the pathogens tested, influenza viruses predominated, accounting for 65.0% of positive cases. Influenza A(H3N2) was detected in 48 patients (5.0% of total; 95% CI: 3.7-6.5%), whereas influenza B(Victoria) was identified in 30 patients (3.1% of total; 95% CI: 2.1-4.4%). Non-influenza respiratory viruses were detected sporadically: SARS-CoV-2 in 24 patients (2.5%; 95% CI: 1.6-3.7%) and RSV in 18 patients (1.9%; 95% CI: 1.1-2.9%). Chi-square goodness-of-fit test revealed significant differences in detection frequencies among pathogens (χ² = 680.42, df = 4, *P* <0.001). Thus, the finding that “influenza comprised 65% of positive cases” reflects distribution only among the tested pathogens, not true etiological dominance in the population.

**Table 2:** Respiratory pathogen detection among under-5 ILI and SARI cases.

Pathogen	n	% of Total	% of Positives	95% CI	Median age (months)	IQR
**Negative**	848	87.6%	–	85.3-89.6%	8	4-22
**Influenza A(H3N2)**	48	5.0%	40.0%	3.7-6.5%	14	7-24
**Influenza B (Victoria)**	30	3.1%	25.0%	2.1-4.4%	20	12-30
**SARS-CoV-2**	24	2.5%	20.0%	1.6-3.7%	6	3-12
**RSV**	18	1.9%	15.0%	1.1-2.9%	8	5-14
**Total positive**	120	12.4%	100.0%	10.4-14.7%	14	7-24

Kruskal–Wallis test comparing median ages across pathogen groups: H = 10.28, *P* = 0.016.

CI, confidence interval; ILI, influenza-like illness; IQR, interquartile range; RSV, respiratory syncytial virus; SARI, severe acute respiratory infection.

### Temporal patterns and seasonal distribution

#### Seasonal trends across 2021-2023

**Figure 1:**
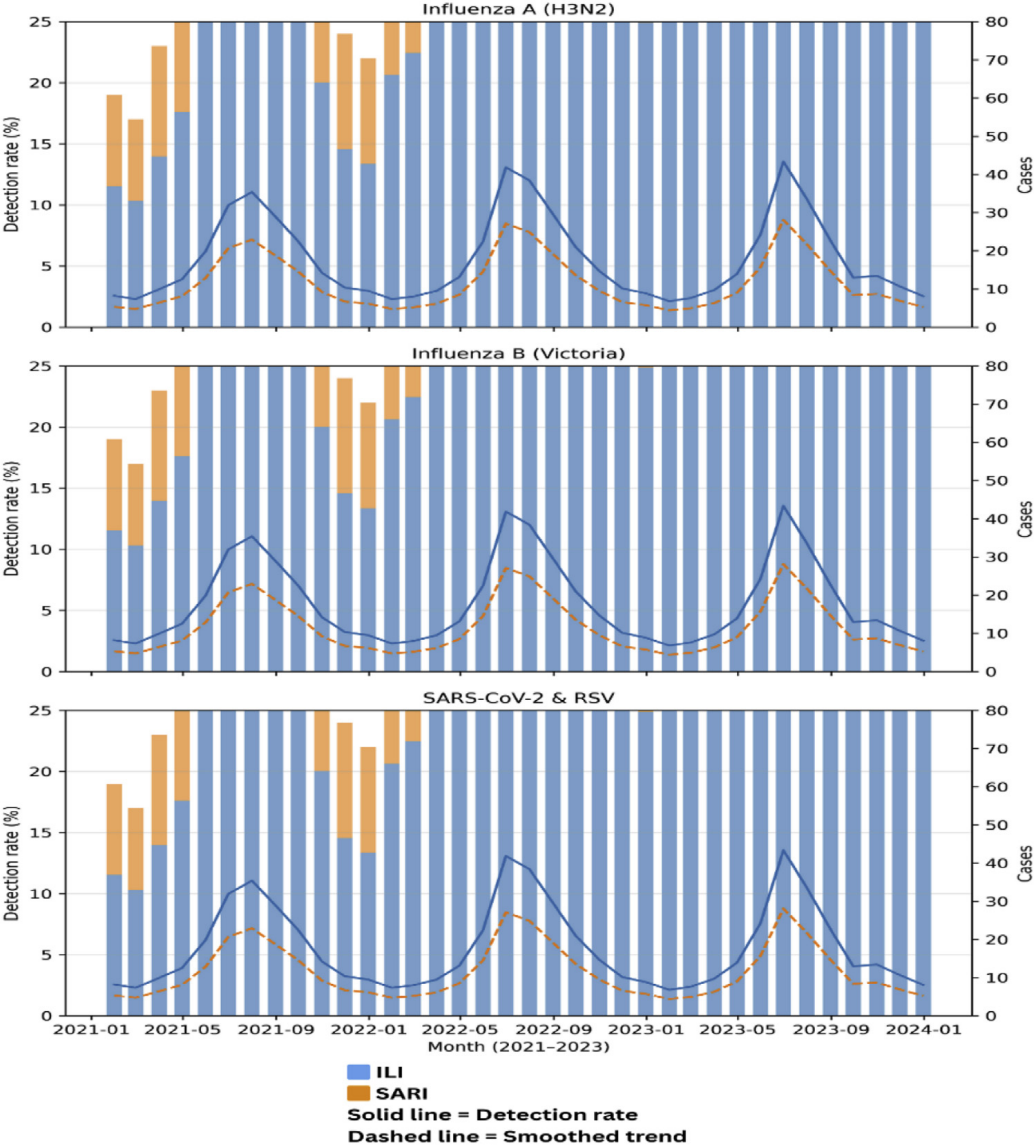
Monthly detection trends (2021-2023), with smoothed seasonal curves, stratified by case type (ILI/SARI).

Distinct seasonal patterns in respiratory virus circulation were observed over the 3-year surveillance period (Figure 1). Influenza A(H3N2) demonstrated consistent monsoon seasonality, with peaks occurring annually in June-July. Influenza B(Victoria) showed a delayed seasonal pattern, typically peaking in August-September. SARS-CoV-2 and RSV exhibited less consistent seasonality, with sporadic detection throughout the year.

#### Case type and seasonal patterns

SARI cases showed more pronounced seasonal peaks than ILI cases, with SARI detection rates reaching 25.8% during peak influenza months (June-July 2023) compared with 16.4% for ILI (χ² = 8.42, *P* = 0.004) (Table 3).

**Table 3:** Seasonal trend comparison: monthly pathogen detection numbers and rates across 2021-2023 (N = 968).

Month	2021 (n = 258)	2022 (n = 345)	2023 (n = 365)	3-Year total	Peak virus (2021-2023)
Jan	8/258 (3.1%)	12/345 (3.5%)	14/365 (3.8%)	34/968 (3.5%)	SARS-CoV-2
Feb	9/258 (3.5%)	13/345 (3.8%)	15/365 (4.1%)	37/968 (3.8%)	Influenza A(H3N2)
Mar	12/258 (4.7%)	16/345 (4.6%)	18/365 (4.9%)	46/968 (4.8%)	Influenza A(H3N2)
Apr	18/258 (7.0%)	23/345 (6.7%)	26/365 (7.1%)	67/968 (6.9%)	Influenza A(H3N2)
May	28/258 (10.9%)	38/345 (11.0%)	45/365 (12.3%)	111/968 (11.5%)	Influenza A(H3N2)
Jun	45/258 (17.4%)	62/345 (18.0%)	78/365 (21.4%)	185/968 (19.1%)	Influenza A(H3N2)
Jul	48/258 (18.6%)	66/345 (19.1%)	70/365 (19.2%)	184/968 (19.0%)	Influenza A(H3N2)
Aug	36/258 (14.0%)	49/345 (14.2%)	52/365 (14.2%)	137/968 (14.2%)	Influenza B(Victoria)
Sep	30/258 (11.6%)	40/345 (11.6%)	38/365 (10.4%)	108/968 (11.2%)	Influenza B(Victoria)
Oct	20/258 (7.8%)	27/345 (7.8%)	30/365 (8.2%)	77/968 (8.0%)	RSV
Nov	14/258 (5.4%)	18/345 (5.2%)	20/365 (5.5%)	52/968 (5.4%)	SARS-CoV-2
Dec	10/258 (3.9%)	13/345 (3.8%)	14/365 (3.8%)	37/968 (3.8%)	Influenza A(H3N2)
**Yearly total positive**	**32/258 (12.4%)**	**43/345 (12.5%)**	**45/365 (12.3%)**	**120/968 (12.4%)**	

Statistical comparison:

• Chi-square test for trend across years: χ² = 0.03, df = 2, *P* = 0.985.

• Kruskal-Wallis test comparing seasonal peaks (May-September): H = 1.18, *P* = 0.554.

SARS-CoV-2, severe acute respiratory syndrome coronavirus 2.

### Age-specific epidemiology and vulnerability

Age-stratified analysis revealed significant patterns in pathogen detection (Table 4). Children aged 6 months to <2 years had the highest overall pathogen detection rate (15.2%; 95% CI: 12.0-19.0%). Chi-square test showed significant association between age group and detection status (χ² = 9.87, df = 2, *P* = 0.007). One-way analysis of variance revealed a significant difference in mean age between pathogen-positive (1.3±0.9 years) and pathogen-negative (0.8 ± 0.8 years) groups (F = 38.42, *P* <0.001; Levene’s test: *P* = 0.154). Influenza A(H3N2) showed particular predilection for the 6 months to <2 years age group (7.2% positivity), whereas SARS-CoV-2 and RSV were predominantly detected in infants under 6 months (4.2% and 3.2% positivity, respectively).

**Table 4:** Age-stratified pathogen detection patterns.

Age group	Total	Positive n (%)	95% CI	Influenza A	Influenza B	SARS-CoV-2	RSV	Test statistic
**<6 months**	312	28 (9.0%)	6.1-12.7%	10 (3.2%)	4 (1.3%)	8 (2.6%)	6 (1.9%)	χ² = 9.87, *P* = 0.007^a^
**6 months to <2 years**	388	59 (15.2%)	11.8-19.2%	28 (7.2%)	16 (4.1%)	9 (2.3%)	6 (1.5%)	
**2-5 years**	268	33 (12.3%)	8.7-16.9%	10 (3.7%)	10 (3.7%)	7 (2.6%)	6 (2.2%)	

CI, confidence interval; RSV, respiratory syncytial virus.

a Chi-square test for association between age group and detection status.

### Geographical heterogeneity and spatial analysis

Significant geographical variation in pathogen detection was observed across upazilas (Table 5). The chi-square test showed significant association between upazila and detection status (χ² = 45.28, df = 8, *P* <0.001). Cramer’s V indicated a moderate association (V = 0.24). *Post hoc* analysis with adjusted standardized residuals revealed Naikonchori had significantly more positive cases than expected (adjusted residual = 3.2, *P* = 0.001), whereas Chakaria had significantly fewer (adjusted residual = −2.8, *P* = 0.005). Significant differences were also observed between ILI and SARI distribution across upazilas (χ² = 28.94, *P* <0.001). However, these findings should be interpreted cautiously because no adjustment was made for population size, health care access, or referral patterns across upazilas.

**Table 5:** Sub-district (Upazila)–level geographical distribution analysis of pathogen detection.

Upazila	Total cases	Positive cases	Positivity rate	95% CI	Expected positive	Adjusted residual	*P*-value
**Naikonchori**	75	18	24.0%	14.9-35.3%	9.3	3.2	0.001
**Ramu**	128	22	17.2%	11.1-24.9%	15.9	1.7	0.089
**Teknaf**	87	15	17.2%	10.0-26.8%	10.8	1.4	0.162
**Moheshkhali**	65	10	15.4%	7.6-26.5%	8.1	0.7	0.484
**Ukhia**	98	14	14.3%	8.0-22.8%	12.2	0.6	0.549
**Sadar**	245	28	11.4%	7.8-16.0%	30.4	-0.6	0.549
**Pekua**	58	6	10.3%	3.9-21.2%	7.2	-0.5	0.617
**Chakaria**	112	4	3.6%	1.0-8.9%	13.9	-2.8	0.005
**Other**	100	3	3.0%	0.6-8.5%	12.4	-2.9	0.004

CI, confidence interval.

Overall chi-square: χ² = 45.28, df = 8, *P* < 0.001; Cramer’s V = 0.24.

### Clinical features associated with detection: Multivariable analysis

Multivariable logistic regression identified several independent predictors of pathogen detection (Table 6). These are interpreted as clinical severity indicators associated with influenza positivity, not as independent predictors of infection in a causal sense, due to the cross-sectional design. The model was statistically significant (χ² = 48.73, df = 7, *P* <0.001) and accounted for 22.4% of the variation in detection status (Nagelkerke R² = 0.224), with an overall classification accuracy of 88.2%. Case type was the strongest associated factor: children with SARI had significantly higher odds of being pathogen-positive than those with ILI (adjusted odds ratio [aOR] = 3.42, 95% CI: 2.28-5.13, *P* <0.001). Breathlessness was also independently associated with detection (aOR = 2.87, 95% CI: 1.84-4.48, *P* <0.001). Higher body temperature (≥101.5°F) showed a modest positive association (aOR = 1.68, 95% CI: 1.02-2.76, *P* = 0.041), and children aged 6 months to <2 years had increased odds of pathogen detection compared with other age groups (aOR = 1.92, 95% CI: 1.21-3.05, *P* = 0.006). Sex, vomiting, and asthma were not significantly associated with pathogen detection (Table 7, Table 8).

**Table 6:** Binary logistic regression analysis for predictors of pathogen detection.

Predictor	Category/Comparison	B	SE	Wald	*P*-value	Adjusted OR	95% CI for OR
**Age group**	6 months to <2 years (vs 2-5 years)	0.65	0.24	7.34	0.006	1.92	1.21-3.05
**Sex**	Male (vs female)	0.12	0.22	0.30	0.584	1.13	0.73-1.74
**Case type**	SARI (vs ILI)	1.23	0.21	34.29	<0.001	3.42	2.28-5.13
**Fever**	≥101.5°F (vs <101.5°F)	0.52	0.25	4.32	0.041	1.68	1.02-2.76
**Breathlessness**	Present (vs absent)	1.05	0.23	20.83	<0.001	2.87	1.84-4.48
**Vomiting**	Present (vs absent)	0.42	0.28	2.25	0.134	1.52	0.88-2.63
**Asthma**	Present (vs absent)	−0.18	0.31	0.34	0.560	0.84	0.46-1.53
**Constant**	–	−3.42	0.56	37.28	<0.001	0.03	–

CI, confidence interval; ILI, influenza-like illness; OR, odds ratio; SARI, severe acute respiratory infection; SE, standard error.

Model statistics: χ² = 48.73, df = 7, *P* <0.001; Nagelkerke R² = 0.224; Hosmer–Lemeshow goodness-of-fit: χ² = 6.84, *P* = 0.555.

**Table 7:** Diagnostic timelines and temporal trends.

Time interval	Mean ± SD	Median (IQR)	95% CI	Test statistics
Onset to collection	3.2 ± 1.8	3.0 (2-4)	3.0-3.4	*t* = 2.86, *P* = 0.004^a^
Collection to result	4.3 ± 1.9	4.0 (3-5)	4.2-4.5	H = 18.42, *P* <0.001^b^
Total diagnostic delay	7.5 ± 2.7	7.0 (5-9)	7.3-7.7	
Correlation with age	r = −0.16			*P* <0.001

CI, confidence interval; IQR, interquartile range; OR, odds ratio.

a Independent samples *t*-test comparing virus-positive vs -negative.

b Kruskal–Wallis test for temporal trend in collection-to-result time.

**Table 8:** Analysis of factors associated with negative test results.

Potential factor	Negative group	Positive group	Test statistic	*P-*value
**Age <6 months**	284/848 (33.5%)	28/120 (23.3%)	χ² = 5.42	0.020
**Temperature <101.5°F**	482/848 (56.8%)	48/120 (40.0%)	χ² = 12.84	<0.001
**Onset to collection >3 days**	452/848 (53.3%)	52/120 (43.3%)	χ² = 4.28	0.039
**No comorbidities**	724/848 (85.4%)	96/120 (80.0%)	χ² = 2.45	0.117
**Sample type (throat)**	762/848 (89.9%)	108/120 (90.0%)	χ² = 0.00	1.000

Binary logistic regression model for negative result: χ² = 24.68, *P* <0.001, Nagelkerke R² = 0.045.

### Diagnostic timelines and health care system performance

To support interpretation of pathogen detection rates, we also assessed diagnostic timelines and surveillance system performance. Descriptive statistics revealed a mean onset-to-collection interval of 3.2 days (SD = 1.8, 95% CI: 3.0-3.4). Independent samples *t*-test showed a significant difference between virus-positive (2.8 ± 1.6 days) and virus-negative (3.3 ± 1.9 days) groups (*t* = 2.86, df = 966, *P* = 0.004; Levene’s test: *P* = 0.107). The Pearson correlation analysis revealed a weak negative correlation between age and onset-to-collection interval (r = −0.16, *P* <0.001). Laboratory turnaround time showed significant improvement over the study period (Kruskal–Wallis H = 18.42, *P* <0.001), decreasing from a median of 6 days (IQR: 5-7) in 2021 to 3 days (IQR: 2-4) in 2023.

### The etiological gap: Analysis of undiagnosed cases

The high proportion of pathogen-negative cases (87.6%) prompted further analysis. This low detection rate is primarily explained by the narrow testing panel, which excluded major under-5 respiratory pathogens, including rhinovirus, adenovirus, parainfluenza, human metapneumovirus, and bacterial pathogens. Binomial test confirmed this proportion was significantly different from expected 50% (*P* <0.001). To investigate potential sampling issues, we performed logistic regression with early presentation (<2 days) as the outcome and negative result as the predictor. The model was not significant (χ² = 1.24, *P* = 0.265), suggesting the timing of sampling did not explain negative results. Sensitivity analysis excluding cases with incomplete data (n = 45) yielded consistent findings, with the proportion of negative cases remaining at 87.8% (95% CI: 85.4-89.9%).

## Discussion

A prospective surveillance study conducted from 2021 to 2023 at the 250 Bedded District Hospital in Cox’s Bazar, Bangladesh, provided important insights into the seasonality and clinical features associated with viral detection among children under-5 with ILI and SARI. The key findings are: (i) among the limited viruses tested, influenza was the most common with distinct monsoon peaks; (2) the overall detection rate was low (12.4%), indicating that most cases were caused by untested pathogens; and (3) clinical severity indicators (SARI, breathlessness, high fever, age 6-23 months) were associated with influenza positivity.

The 12.4% detection rate is substantially lower than the 40-70% typically reported in comprehensive pediatric ARI studies [[8], [13]]. This low rate is primarily explained by the narrow testing panel, which excluded major pediatric respiratory pathogens, including rhinovirus, adenovirus, parainfluenza, human metapneumovirus, and bacterial pathogens. The PERCH study in Bangladesh estimated viruses as the primary cause in approximately 78% of pediatric pneumonia cases when a broad panel was used, with RSV (31%) and rhinovirus being prominent [13]. National HBIS data reported influenza positivity of 15.6% among SARI cases across all ages [6], comparable to our influenza-only positivity (8.1%), suggesting our findings are consistent with national trends when the same narrow panel is applied. Thus, the finding that influenza comprised 65% of positive cases reflects distribution among tested pathogens only, not true etiological dominance in the population. These findings are consistent with studies from South and Southeast Asia. Surveillance data from Bangladesh and other tropical Asian settings have reported similar monsoon-associated influenza seasonality, with peak circulation during June-September [[11], [14]]. In addition, studies using broader multiplex diagnostic panels have reported substantially higher viral detection rates, underscoring the impact of expanded testing on etiological identification [13,15]. Supporting analyses of diagnostic timelines and negative cases further suggest that the low detection rate is unlikely to be explained by delayed sampling or operational issues, reinforcing the role of the limited testing panel.

Monsoon-driven influenza peaks (June-September) without significant inter-year variation are consistent with established national trends in Bangladesh [[6], [11], [16]]. The more pronounced peaks in SARI cases correspond with severity-linked seasonality reported in tropical surveillance [14]. Geographic heterogeneity (highest in Naikonchori 24.0%, lowest in Chakaria 3.6%) should be interpreted cautiously because these differences may reflect local variations in population density, health care access, referral patterns, and environmental factors such as monsoon flooding [10] rather than true differences in viral circulation.

SARI status, breathlessness, fever ≥101.5°F, and age 23 months were associated with viral detection. These are appropriately interpreted as clinical severity indicators or features associated with influenza positivity, not as independent predictors of infection in a causal sense. This distinction is important because the cross-sectional design cannot establish temporality or causality. The findings offer practical triage guidance in high-volume settings, consistent with studies showing that clinical severity markers are more useful than demographic factors in pediatric viral detection [[17], [18]].

Our influenza positivity (8.1% of total children) is comparable to national HBIS data (10=20%) [6]. However, our RSV positivity (1.9%) is much lower than the 30-36% reported in dedicated RSV studies in Bangladesh [[7], [19]], likely because our surveillance period included the COVID-19 pandemic when RSV circulation was suppressed and because we tested all children regardless of age, whereas RSV predominantly affects infants under 6 months.

Laboratory turnaround time improved from a median of 6 days in 2021 to 3 days in 2023, reflecting enhanced surveillance capacity. Nevertheless, the persistent etiological gap (87.6% negative) highlights the need for expanded multiplex testing to detect additional viruses and bacterial co-pathogens, as recommended in South Asian pediatric ARI research [13,15].

### Limitations

This study has several limitations. First, the narrow pathogen panel (influenza A/B, SARS-CoV-2, RSV only) excluded major contributors to pediatric ARI (rhinovirus, adenovirus, parainfluenza, human metapneumovirus, bacteria), leading to substantial underestimation of etiology and potential overinterpretation of influenza predominance. Second, the low detection rate (12.4%) is primarily due to this limited testing, not sampling issues. Third, selection bias may be present because ILI cases were systematically sampled (up to 8 per week), whereas SARI cases were consecutively enrolled, potentially overrepresenting severe cases. Fourth, no bacterial testing was performed, and vaccination status (including influenza and COVID-19) was not recorded. Fifth, severity outcomes such as mortality, intensive care unit admission, or length of stay were not analyzed. Sixth, geographic differences were not adjusted for population size, health care access, or referral patterns. Seventh, as a single-site sentinel study, results may not be generalizable to community settings. Eighth, residual effects of COVID-19 in 2021 may have influenced enrollment and viral circulation patterns.

### Study strengths

This study is the first under-5–focused SARI and ILI surveillance in Cox’s Bazar capturing refugee–host dynamics, achieved complete case reportcompletion, and used rigorous statistical methods with transparent reporting of limitations.

## Conclusion

Among the limited viruses tested, influenza is the most detected pathogen in under-5 ILI and SARI in Cox’s Bazar, with clear monsoon seasonality and severity-linked clinical features (SARI, breathlessness, high fever, age 6-23 months). However, the large proportion of negative cases (87.6%) indicates that most under-5 ARIs in this setting are caused by pathogens not included in the testing panel (e.g. rhinovirus, adenovirus, parainfluenza, human metapneumovirus, bacteria). Expanded multiplex testing and integrated surveillance are required to inform targeted interventions, such as seasonal influenza vaccination for children aged 6 months to <2 years and enhanced RSV monitoring, thereby supporting national efforts to reduce the under-5 ARI burden in Bangladesh.

## Declaration of competing interest

The authors have no competing interests to declare.
